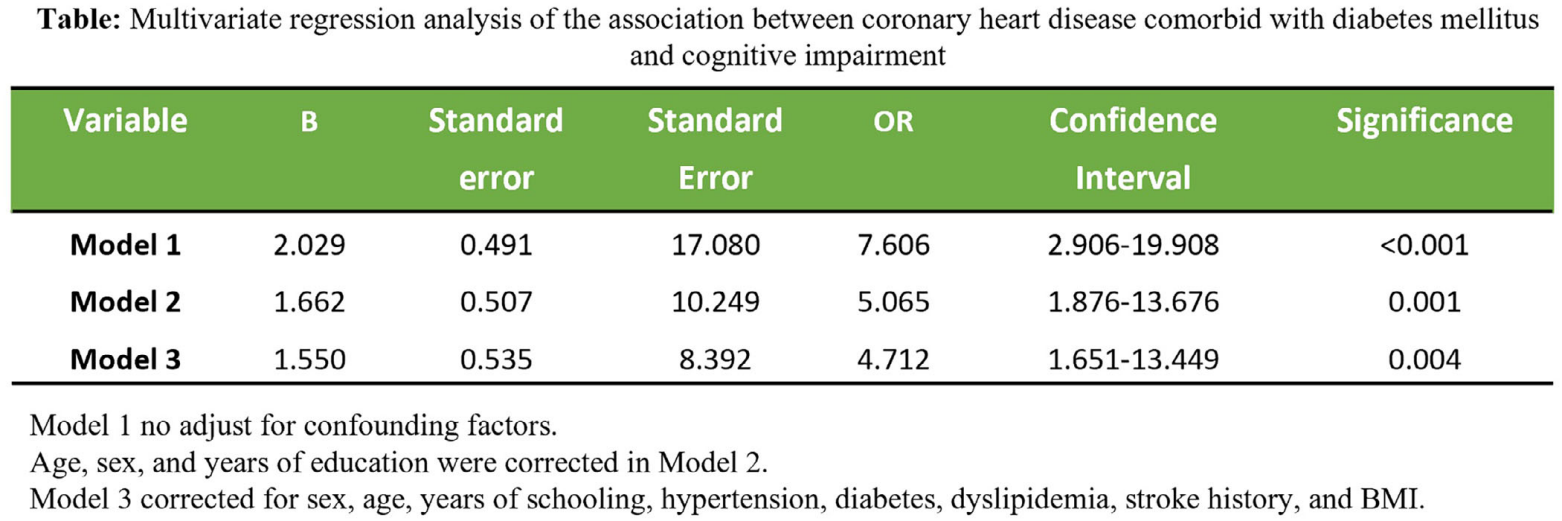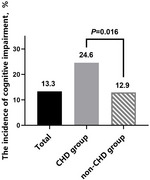# The Correlation Between Coronary Heart Disease and Cognitive Impairment in Middle‐aged and Elderly Populations in Rural Xi'an: A Community‐Based Cross‐Sectional Study

**DOI:** 10.1002/alz70860_099206

**Published:** 2025-12-23

**Authors:** Meng Wei, Qiumin Qu, Suhang Shang, Kang Huo

**Affiliations:** ^1^ First Affiliated Hospital of Xi‘an Jiaotong University, Xi‘an, Shaanxi, China; ^2^ The First Affiliated Hospital of Xi'an Jiaotong University, Xi'an, Shaanxi, China

## Abstract

**Background:**

Cardiovascular disease (CVD) and cognitive impairment are significant public health challenges, both conditions share common risk factors like hypertension and obesity. Previous studies indicated a link between CVD and cognitive impairment, but focused on hospitalized patients. This research aims to investigate the correlation between coronary heart disease (CHD) and cognitive impairment in rural populations aged 40 and above.

**Method:**

From October 2014 to March 2015, residents aged 40 and above from a village in Huyi District, Xi'an, were selected as study subjects. Data on demographics, lifestyle habits, medical history, family history, physical examinations, and biochemical tests were collected. Participants were categorized into those with and without a history of CHD. Cognitive function was assessed using the Mini‐Mental State Examination (MMSE), with scores below the cutoff (illiteracy ≤ 17; primary school ≤ 20; junior high school and above ≤ 24) defined as cognitive impairment. Chi‐square tests were used to compare the prevalence of cognitive impairment between the CHD and non‐CHD groups. Multivariate logistic regression was employed to adjust for confounding factors in analyzing the relationship between CHD and cognitive impairment.

**Result:**

(1) A total of 1,833 subjects were included in the analysis, comprising 735 males (40.1%) and 57 individuals with CHD (3.1%).

(2) Among them, 234 participants (13.3%) met the criteria for cognitive impairment.

(3) Univariate analysis showed a higher prevalence of cognitive impairment in the CHD group compared to the non‐CHD group (24.6% vs 12.9%, *p* = 0.016). (Fig)

(4) Unadjusted binary logistic regression indicated a positive correlation between CHD and cognitive impairment (OR = 2.199[95% CI, 1.185‐4.084], *p* = 0.013).

(5) However, after adjusting for confounding factors such as gender, age, education level, hypertension, diabetes, dyslipidemia, stroke history, and BMI, the association between CHD and cognitive impairment was not statistically significant (OR=1.265 [95% CI, 0.656‐2.441], *p* = 0.483).(Table)

(6) The prevalence of cognitive impairment significantly increases in patients with coronary heart disease combined with diabetes (OR=4.712, [95% CI 1.651‐13.44], *p* = 0.004).

**Conclusion:**

This study did not establish a direct association between CHD and cognitive impairment but confirmed that CHD combined with diabetes significantly increases the risk of cognitive impairment. Future prospective studies with larger sample sizes should be conducted to further confirm the relationship between the two.